# Subclinical Hypothyroidism Is Associated with Increased Risk for Cancer Mortality in Adult Taiwanese—A 10 Years Population-Based Cohort

**DOI:** 10.1371/journal.pone.0122955

**Published:** 2015-04-01

**Authors:** Fen-Yu Tseng, Wen-Yuan Lin, Chia-Ing Li, Tsai-Chung Li, Cheng-Chieh Lin, Kuo-Chin Huang

**Affiliations:** 1 Department of Internal Medicine, National Taiwan University Hospital, Taipei, Taiwan; 2 Family Medicine, National Taiwan University Hospital, Taipei, Taiwan; 3 Department of Family Medicine, China Medical University Hospital, Taichung, Taiwan; 4 Medical Research, China Medical University Hospital, Taichung, Taiwan; 5 School of Medicine, China Medical University, Taichung, Taiwan; 6 Graduate Institute of Clinical Medicine Science, China Medical University, Taichung, Taiwan; 7 Graduate Institute of Biostatistics, China Medical University, Taichung, Taiwan; 8 Department of Internal Medicine, National Taiwan University College of Medicine, Taipei, Taiwan; 9 Institute of Health Care Administration, College of Health Science, Asia University, Taichung, Taiwan; Uppsala University, SWEDEN

## Abstract

**Background:**

The association between subclinical hypothyroidism (SCH) and cancer mortality is seldom discussed.

**Methods:**

A total of 115,746 participants without thyroid disease history, aged 20 and above, were recruited from four nationwide health screening centers in Taiwan from 1998 to 1999. SCH was defined as a serum thyroid-stimulating hormone (TSH) level of 5.0–19.96 mIU/L with normal total thyroxine concentrations. Euthyroidism was defined as a serum TSH level of 0.47–4.9 mIU/L. Cox proportional hazards regression analyses were used to estimate the relative risks (RRs) of death from cancer for adults with SCH during a 10-year follow-up period.

**Results:**

Among 115,746 adults, 1,841 had SCH (1.6%) and 113,905 (98.4%) had euthyroidism. There were 1,532 cancer deaths during the 1,034,082 person-years follow-up period. Adjusted for age, gender, body mass index, diabetes, hypertension, dyslipidemia, smoking, alcohol drinking, betel nut chewing, physical activity, income, and education level, the RRs (95% confidence interval) of cancer deaths among subjects with SCH versus euthyroid subjects were 1.51 (1.06 to 2.15). Cancer site analysis revealed a significant increased risk of bone, skin and breast cancer among SCH subjects (RR 2.79, (1.01, 7.70)). The risks of total cancer deaths were more prominent in the aged (RR 1.71, (1.02 to 2.87)), in females (RR 1.69 (1.08 to 2.65)), and in heavy smokers (RR 2.24, (1.19 to 4.21)).

**Conclusions:**

Subjects with SCH had a significantly increased risk for cancer mortality among adult Taiwanese. This is the first report to demonstrate the association between SCH and cancer mortality.

## Introduction

The association between thyroid hormone and tumorigenesis has been discussed for more than 30 years. In *in vitro* studies, it was reported that triiodothyronine (T3) facilitated chemical carcinogenesis [1]. Removal of T3 or T4 from serum eliminated X-ray induced neoplastic transformation, while adding T3 re-established the expected frequency of transformation [2]. Theoretically, deficit or excess of thyroid hormone may alter the hormonal milieu thus initiate or promote tumor growth. However, no consensus has been reached concerning the effects of different thyroid function status on cancer incidence or mortality.

Subclinical hypothyroidism (SCH) is defined as normal serum thyroxine (T4) level with elevated thyroid stimulating hormone (TSH). The proposed adverse consequences of SCH include systemic hypothyroid symptoms, psychiatric symptoms, progression to overt hypothyroidism, and hypercholesterolemia [3–4]. Effects of SCH on cardiovascular events, all-cause mortality, and cardiovascular death have been discussed repeatedly in literature [5–11]. However, only a few reports discuss the associations between SCH and cancer mortality. Our previous study reported increased risk for all-cause and cardiovascular disease (CVD) mortality in adults with SCH [9]. In this study, we aimed to evaluate the impact of SCH on cancer mortality in a large Taiwanese cohort.

## Patients and Methods

### Subjects and measurements

The data were collected from four private nationwide MJ Health Screening Centers in Taiwan. The registered health practitioners in these centers provide a multidisciplinary team approach of health assessment for their members. Most of the members undergo health examinations every 3–4 years voluntarily and approximately 30% of them will receive the same health check-up every year. All the 124,456 participants who received a health exam at MJ centers during the period between 1998 and 1999, aged 20 years and above, were recruited into this study. Nine hundred fifty-three participants (0.8%) who had a history of thyroid disease with medication treatment and 3,310 participants (2.6%) with missing TSH or total T4 level at entry were excluded. SCH was defined as a serum TSH level of 5.0–19.96 mIU/L with normal total T4 concentrations (57.9–154.4 nmol/L). Euthyroidism was defined as a serum TSH level of 0.47–4.9 μIU/mL [9]. Therefore, participants with serum TSH level ≥ 20 mIU/L or < 0.47 mIU/L were also excluded (n = 4,447). Finally, 115,746 participants were included for analyses in the study as in our previous study [9].

The age and gender distribution of our study was similar to the Taiwan national population. [12]. Deaths were ascertained by computer linkage to the national death registry (death certificates created by the Department of Health, Taiwan) using ID numbers. All deaths that occurred between study entry (year 1998) and December 2008 were included. Deaths with the International Classification of Disease, Ninth Revision, Clinical Modification (ICD-9-CM) codes 140 to 208 were classified as cancer deaths, while individual site-specific cancers were further classified by the ICD-9-CM codes.

### Anthropometric index and laboratory assays

The anthropometric characteristics, blood pressure (BP), plasma glucose, total cholesterol (TCHOL), high-density lipoprotein cholesterol (HDL-C), and triglycerides were measured as described in the previous report [13]. Thyroid function (TSH and Total T4) was also measured (ABBOTT AxSYM, Illinois, U.S.A.). The coefficients of variation were 3.6%~4.3% at level 2.837~3.419 mIU/L and 8.1%~8.8% at level 15.32~19.727 mIU/L for the precision of TSH assay, and were 2.8%~3.6% at level 7.9~8.5μg/dL for that of total T4. In brief, trained staff measured height (measured to nearest 0.1cm) and weight (measured to nearest 0.1kg). Body mass index (BMI) was calculated as weight (kg) divided by height squared (m^2^). All anthropometric measurements were performed twice, and the mean value was used for analysis. The BP was measured in the right arm using an appropriately-sized cuff and a standard mercury sphygmomanometer while participants were in a seated position. Blood was drawn with minimal trauma from an antecubital vein in the morning after a 12-hour overnight fast. Diabetes was defined as a fasting glucose ≥ 126 mg/dL and/or history of diabetes and taking oral hypoglycemic agents or insulin. Hypertension was defined as systolic BP ≥ 140 mmHg, and/or diastolic BP ≥ 90 mmHg, and/or history of hypertension or taking anti-hypertensive drugs. Dyslipidemia was defined as TCHOL ≥ 200mg/dL and/or triglycerides ≥ 150 mg/dL and/or HDL-C < 40 mg/dL in men and <50 mg/dL in women and/or history of dyslipidemia and taking anti-dyslipidemia drugs. Ethic approval for patient recruitment and data analyses was obtained from the MJ Research Foundation Review Committee in Taiwan. Written informed consents have been obtained from all the 124,456 participants.

### Questionnaire

Cigarette smoking, alcohol intake, betel nut chewing, and physical activity histories were recorded for each subject using questionnaires as in previous reports [14–15]. Current, former, or never users for smoking, alcohol intake, and betel nut chewing were defined as those who reported the current use, any prior use, or never use of these substances, respectively, at baseline survey. The cumulative exposure to smoking was assessed by recording the duration (years) and quantity (number of cigarettes/day). Former users were also asked for their age at quitting. Cumulative pack-years of smoking were calculated as smoking-years multiplied by average daily cigarette use divided by 20. Cumulative pack-years for smokers were categorized into two groups (Low: <10 pack-years; High: ≥ 10 pack-years), so smoking status was categorized as none (0 pack-years), low (0~9.9 pack-years), and high (≥ 10 pack-years). Physical activity was classified into three levels: none to mild (exercise less than one hour per week), moderate (exercise one to four hours per week), and vigorous (exercise more than five hours per week) physical activity. Income status was sub-divided into three levels: low (< USD 12,500/year), middle (12,500–37,500/year), and high (>37,500/year). Education was also sub-divided into three levels: low (elementary school and below), middle (junior and senior high school), and high (college/university and above).

### Statistical analysis

**Fig 1:**
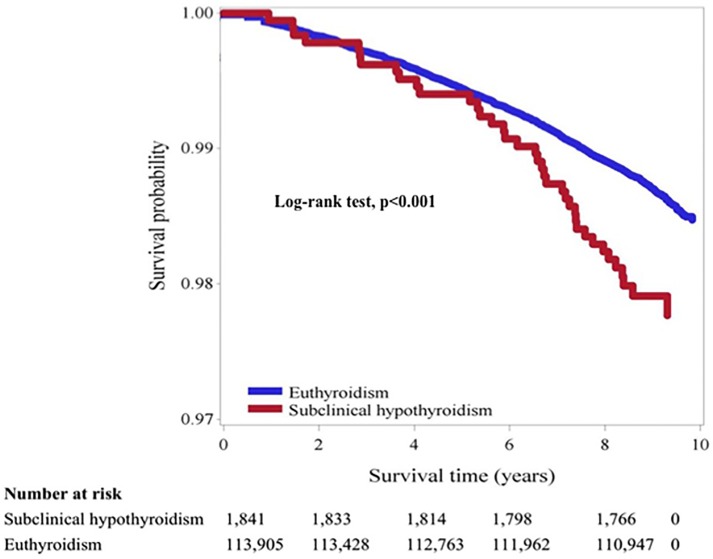
Comparison of cancer morality in subjects with subclinical hypothyroidism or euthyroidism.

The data were presented as the means and standard deviation for continuous variables. Student’s *t* test for unpaired data was used for the comparison of mean values between two groups. Proportions and categorical variables were tested by the χ^2^ test. The unadjusted Kaplan-Meier survival curves of cancer mortality for SCH and euthyroid subjects are shown in Fig 1. Cox proportional hazards regression analyses adjusted for potential confounders were used to estimate the relative risks (RRs) for cancer mortality [16–17]. We adjusted the covariates according to the cancer risk factors, or on the basis of their relationship with either SCH (predictor) or cancer death (outcome) in univariate analysis (p < 0.05). For example, age and gender are strongly associated with mortality, so we adjusted these two covariates in our Cox proportional hazards regression analyses. Lifestyle and socioeconomic status such as physical activity, income level, and education level are risk factors for mortality, so we adjusted these covariates. Smoking, alcohol drinking, betel nut chewing, diabetes, hypertension, obesity, and dyslipidemia are associated with increased risk of cancer and/or cancer mortality, so we also adjusted these covariates. The RRs were calculated and presented with 95% confidence interval (CI). Participants with missing covariate data were excluded in the Cox proportional hazards regression analyses. Competing risk approached by cumulative incidence competing risk estimate were analyzed. Site-specific cancer deaths among subjects with SCH or euthyroidism were compared. We performed stratified analysis by gender, age, TSH level, or smoking status at entry for the association between SCH and risk of cancer death. All analyses were performed using the PC version of SPSS statistical software (17th version, SPSS Inc., Chicago, IL, USA).

### Role of funding source

The funding source had no role in the study design, data collection, data analysis, data interpretation, writing of the report, or in the decision to submit the paper for publication. All authors had full access to all the data in the study and KCH had final responsibility for the decision to submit for publication.

## Results

At the baseline survey, there were 1,841 (1.6%) subjects with SCH and 113,905 (98.4%) subjects with euthyroidism. The prevalence of SCH was 0.9% (512/54,983) in men and 2.2% (1,329/60,763) in women, respectively. Subjects with SCH tended to be females (72.2% vs 52.2%), were older (47.1±14.1 vs 42.9±13.9 y/o), and had higher BMI, BP, fasting glucose, TCHOL, HDL-C, and triglycerides levels than subjects with euthyroidism. Those with SCH also differed with euthyroid subjects in smoking, alcohol consumption, betel nut chewing, income, and education (Table 1).

**Table 1:** Baseline characteristics of subjects with subclinical hypothyroidism or euthyroidism.

	Subclinical hypothyroidism	Euthyroidism	*P* value
	(n = 1,841)	(n = 113,905)	
Age (years)^1^	47.1±14.1	42.9±13.9	<0.001
Male (n, %)^2^	512 (27.8%)	54,471 (47.8%)	<0.001
BMI (kg/m^2^)^1^	23.5±3.7	23.1±3.5	<0.001
Systolic BP (mmHg)^1^	124.2±23.0	120.4±20.6	<0.001
Diastolic BP (mmHg)^1^	74.7±12.8	73.4±12.7	<0.001
Fasting glucose (mmol/L)^1^	5.62±1.62	5.48±1.29	<0.001
TCHOL (mmol/L)^1^	5.36±1.04	5.21±1.00	<0.001
Triglycerides (mmol/L)^1^	1.54±1.13	1.41±1.19	<0.001
HDL-C (mmol/L)^1^	1.29±0.41	1.26±0.40	<0.001
TSH (mIU/L)	7.07±2.44	1.56±0.79	<0.001
T4 (nmol/L)	93.1±17.3	99.9±19.1	<0.001
Diabetes (n, %)^2^	122 (6.6%)	5430 (4.8%)	<0.001
Hypertension (n, %)^2^	516 (28.0%)	22,728 (20.0%)	<0.001
Smoking (n = 110,386)^2^			<0.001
Never	1,411(81.1%)	77,045(70.9%)	
Former	100(5.7%)	7,148(6.6%)	
Current	230(13.2%)	24,452(22.5%)	
Alcohol consumption (n = 106,136)^2^			<0.001
Never	1,360(82.7%)	82,023(78.5%)	
Former	60(3.6%)	3,612(3.5%)	
Current	225(13.7%)	18,856(18.0%)	
Betel nut chewing (n = 109,332)^2^			<0.001
Never	1,615(93.8%)	96,312(89.5%)	
Former	44(2.6%)	5,380(5.0%)	
Current	63(3.7%)	5,918(5.5%)	
Physical activity (n = 109,003)^2^			0.252
None/mild	872(50.7%)	53,639(50.0%)	
Moderate	577(33.5%)	37,828(35.3%)	
Vigorous	271(15.8%)	15,816(14.7%)	
Income (n = 108,593)^2^			<0.001
Low	1,035(61.6%)	51,278(48.0%)	
Middle	568(33.8%)	48,833(45.7%)	
High	76(4.5%)	6,803(6.4%)	
Education (n = 112,286)^2^			<0.001
Low	634(35.7%)	25,355(22.9%)	
Middle	645(36.3%)	40,270(36.4%)	
High	497(28.5%)	44,885(40.7%)	

^1^Student t-test was used for comparing mean values of continuous variables between groups; data were shown as the mean ± SD; log transformation were used for normal distribution

^2^Pearson chi-square test was used for categorical data; data were shown as percentage

Abbreviations: BMI, body mass index; BP, blood pressure; TCHOL, total cholesterol; HDL-C, high-density lipoprotein cholesterol; TSH, thyroid-stimulating hormone. T4: thyroxine.

There were 3,669 deaths during the 10 years of follow-up. Among them, 1,532 deaths were due to cancer (38 in SCH subjects and 1494 in euthyroid subjects). The cancer death rate among SCH subjects was significantly higher than that among euthyroid subjects (2.06% vs 1.31%, Chi-Square p = 0.0051). The comparison of cancer mortality in subjects with subclinical hypothyroidism or euthyroidism was shown in Fig 1. The unadjusted Kaplan-Meier survival curves revealed significantly more cancer deaths among subjects with SCH (Log-rank test, p<0.001).

Competing risk approached by cumulative incidence competing risk estimate were done in Table 2. After adjustment for age, gender, BMI, diabetes, hypertension, dyslipidemia, smoking, alcohol drinking, physical activity, income, and education level, the RRs of cancer deaths among subjects with SCH versus euthyroid subjects was 1.51 (95% CI, 1.06 to 2.15). To clarify the effects of potential diseases on mortality, we excluded subjects who died during the first 3 years of follow-up. After excluding study subjects who died during the first 3 years of follow-up, compared with subjects with euthyroidism, the adjusted RRs for cancer death for subjects with SCH were 1.61 (95% CI, 1.09 to 2.61) (Table 2).

**Table 2:** Relative risks (95% confidence interval) of subclinical hypothyroidism for cancer mortality using Cox proportional hazards regression analyses, unadjusted or adjusted for potential confounders.

	Death (n)	Cancer Mortality Rate Per 1,000 Person-Years	Unadjusted	Adjusted^§^
			Cancer mortality relative risk	
			(95% confidence interval)	
Euthyroidism				
1998/1999-2008	1,494	1.468	1.00(reference)	1.00(reference)
2001/2002-2008^#^	1,171	1.152	1.00(reference)	1.00(reference)
SCH				
1998/1999-2008	38	2.322	1.59(1.15 to 2.19)^†^	1.51(1.06 to 2.15)*
2001/2002-2008^#^	31	1.899	1.77(1.26 to 2.49) ^†^	1.61(1.09 to 2.61)*

*p <0.05;

^†^p <0.01;

^§^Adjusted RR: adjusted for age, gender, body mass index, diabetes, hypertension, dyslipidemia, smoking, alcohol drinking, betel nut chewing, physical activity status, income, and education level

^#^Excluded those who died during first 3 years follow-up (only deaths that occurred after more than 3 years of follow-up were included).

Cancer mortality was determined with a competing risk approach by cumulative incidence competing risk estimate with adjustment for non-cancer mortality. The models used Cox proportional hazards regression analyses adjusted for potential confounders.

We further analyzed the difference of cancer deaths between SCH and euthyroid subjects according to the sites of the cancer. Subjects with SCH had significant higher risks of death from bone, skin and breast cancer than euthyroid subjects (RR 2.79, 95% CI 1.01 to 7.70) (Table 3).

**Table 3:** Site-specific cancer deaths of subjects with SCH or euthyroidism.

	SCH	Euthyroidism	Adjusted relative risk (95% C.I.)^b^
	(n)	(n)	
Total	1,841	113,905	
All cancer deaths	38	1,494	1.51 (1.06, 2.15)^c^
Oral cavity (140–149)^a^	2	103	1.68 (0.41, 6.85)
Digestive (150–159)^a^	18	696	1.64 (1.00, 2.70)
Respiratory (160–165)^a^	11	333	1.81 (0.89, 3.67)
Bone, skin, breast (170–176)^a^	4	76	2.79 (1.01, 7.70)^c^
Genitourinary (ICD 179–189)^a^	1	133	0.34 (0.05, 2.41)
Others and unspecified (190–199)^a^	1	53	1.21 (0.17, 8.84)
Hematopoietic (200–208) ^a^	1	100	0.68 (0.10, 4.93)

SCH: subclinical hypothyroidism

n: subject number

C.I.: confidence interval

^a^: ICD code

^b^: relative risks of deaths among SCH subjects versus euthyroid subjects

^c^: adjusted for age, gender, body mass index, diabetes, hypertension, dyslipidemia, smoking, alcohol drinking, betel nut chewing, physical activity status, income, and education level. P<0.05

There were no significant interactions (P> 0.05) between SCH/euthyroidism groups and age group/gender group/TSH status/smoking status for predicting the risk of cancer mortality. However, gender, age, serum TSH level, and smoking status might be important risk factors for cancer mortality. We therefore stratified these groups and presented the results in Table 4. Compared to subjects with euthyroidism, both male and female subjects with SCH had increased crude RRs of cancer deaths. The adjusted RR of cancer deaths among female SCH subjects persisted as statistically significant (RR 1.69, 95% CI 1.08 to 2.65) than female euthyroid subjects (Table 4). Increasing risks of cancer death was more prominent at aged subjects. In subjects with age equal or older than 65 years, the adjusted RR of cancer death was 1.71 (95% CI 1.02 to 2.87) when compared SCH subjects to euthyroid subjects (Table 4). Subjects with TSH level between 5.0 to 9.99 μIU/mL had significantly higher risks of cancer death than euthyroid subjects (adjusted RR 1.61, 95% CI 1.12 to 2.31) (Table 4). High-dose smokers had higher RRs for cancer mortality than the never smokers or low-dose smokers. The adjusted RR of cancer deaths in SCH subjects versus euthryoid subjects among those with high-dose smokers was 2.24 (95% CI: 1.19 to 4.21) (Table 4).

**Table 4:** Relative risks (95% confidence interval) of subclinical hypothyroidism for cancer mortality stratified by gender, age, TSH level, and smoking status using Cox proportional hazards regression analyses adjusted for potential confounders.

Variables	Death (n)	Number at risk	Cancer Mortality Rate Per 1,000 Person-Years	Unadjusted	Adjusted^§^
		(n)		Cancer mortality relative risk	
				(95% confidence interval)	
Male					
Euthyroid	901	54,471	1.860	1.00(reference)	1.00(reference)
SCH	15	512	3.351	1.81 (1.09 to 3.02)*	1.32 (0.75 to 2.34)
Female					
Euthyroid	593	59,434	1.112	1.00(reference)	1.00(reference)
SCH	23	1,329	1.935	1.74 (1.15 to 2.64)^†^	1.69 (1.08 to 2.65)*
Age < 65 years					
Euthyroidism	883	104,326	0.943	1.00(reference)	1.00(reference)
SCH	19	1,622	1.303	1.38(0.88 to 2.18)	1.36(0.84 to 2.20)
Age ≥ 65 years					
Euthyroidism	611	9,579	7.550	1.00(reference)	1.00(reference)
SCH	19	219	10.660	1.43(0.91 to 2.26)	1.71(1.02 to 2.87)*
TSH (μIU/mL)					
0.47–4.99	1,494	113,905	1.468	1.00 (reference)	1.00(reference)
5.0–9.99	36	1,635	2.482	1.69 (1.22 to 2.36) ^†^	1.61 (1.12 to 2.31)*
10–19.96	2	206	1.076	0.73 (0.18 to 2.93)	0.80 (0.20 to 3.20)
Smoking: Never					
Euthyroidism	903	83,275	1.210	1.00(reference)	1.00(reference)
SCH	24	1,528	1.763	1.46(0.97 to 2.19)	1.32(0.85 to 2.06)
Smoking: < 10PY					
Euthyroidism	170	15,161	1.257	1.00(reference)	1.00(reference)
SCH	2	158	1.421	1.41(0.35 to 5.71)	1.28(0.32 to 5.20)
Smoking: ≥ 10PY					
Euthyroidism	421	15,469	3.088	1.00(reference)	1.00(reference)
SCH	12	155	8.968	2.91(1.64 to 5.17)^‡^	2.24(1.19 to 4.21)*

*p <0.05;

^†^p <0.01;

^‡^p <0.001

^§^Adjusted RR: adjusted for age, gender, body mass index, diabetes, hypertension, dyslipidemia, smoking, alcohol drinking, betel nut chewing, physical activity status, income, and education level.

## Discussion

In literature reviews, the prevalence of SCH was between 4% and 20%, higher in the aged and in women [18–21]. A previous study in Southern Taiwan reported a SCH prevalence of 1.5% in women and 1.7% in men [22]. Comparable to that study, the prevalence of SCH was 0.9% in men, 2.2% in women, and 1.6% in total in this cohort.

The status of BMI, DM, hyperlipidemia, or hypertension in SCH varied in previous literature. BMI has been reported to be positively correlated with serum TSH levels [23–24], but some other studies reported no difference in BMI between subjects with SCH or euthyroid status [25–28]. Thyroid disorders may cause dyslipidemia. It was reported that levels of TCHOL, triglyceride, low-density lipoprotein cholesterol (LDL-C) elevate as thyroid function declines [19, 25, 27–30]. The association between SCH and hypertension was controversial in previous literature. Some, but not all, studies demonstrated a higher prevalence of hypertension in SCH subjects [25, 31–32]. Previous literature demonstrated no difference in levels of fasting glucose or hemoglobin A1C [27–28], and the prevalence of diabetes mellitus [5, 25–26] between the SCH and euthyroid subjects. Obesity, hypertension, diabetes, and dyslipidemia had been associated with cancer incidence or mortality [33–40]. Smoking, alcohol consumption and betel nut chewing were also known as risk factors for lung, pancreatic, kidney, colorectal, or oral-esophageal cancer, respectively [41–43]. Our analysis revealed that participants with SCH were older, had higher BMI, BP, fasting glucose, TCHOL, triglyceride levels, and higher rates of smoking, alcohol consumption, and betel nut chewing than those with euthyroidism. Our analysis revealed that subjects with SCH had significantly more risk factors for cancer than euthyroid subjects.

The association between hypothyroidism and cancer risk is controversial. Only a few studies discuss the association between SCH and cancer risks. Hellevik et al. reported that hypothyroidism was not associated with cancer risk [44]. It was observed that breast cancer might occur more frequently in hypothyroid women [45], while some researchers reported lower risk, delayed onset, smaller tumor size, or fewer metastases of breast cancer in hypothyroid patients [46–47]. Hoffman et al. reported that use of thyroid supplements does not increase the risk of developing breast cancer [48], while others reported that the risks of cancer increased in patients who received thyroid supplements for hypothyroidism [49–50].

Debates also exist concerning the influence of hypothyroidism and SCH on cancer prognosis. Goldman et al. reported that hypothyroid women had no significant increase in standardized mortality ratio of cancer [51]. Hercbergs et al. reported a case with metastatic lung cancer having no tumor recurrence after a myxedema coma episode [52]. Metso et al. reported that RAI-treated hyperthyroidism increased overall, CVD and cancer mortality, but the development of hypothyroidism reduced mortality significantly [53]. In patients with renal cell carcinoma treated by sunitinib or sorafenib, development of SCH or hypothyroidism was identified as an independent predictor of survival [54–55]. In patients with head and neck tumors, iatrogenic hypothyroidism secondary to regimens for cancers was associated with longer survival [56]. In a literature review, the association between hypothyroidism and cancer prognosis had been discussed in lung cancer, various solid tumors, breast cancer, renal cell carcinoma, anaplastic thyroid cancer, and high-grade gliomas, etc [57]. In our study, compared with euthyroid subjects, the relative risks of cancer deaths in SCH subjects significantly increased, especially in bone, skin, or breast cancer (ICD-9-CM codes 170–176). The effects of SCH on cancer mortality persisted even if we excluded mortality within the first 3 years and after adjustment with demographic, anthropometric, clinical, and socioeconomic characteristics. Our analysis suggests that SCH is an independent risk factor for cancer mortality. This observation was never reported before.

SCH increased cancer mortality in both genders, but the effect of SCH on cancer death was less statistically significant in men. The effects of SCH may vary in different age groups. Our previous study reported a more significantly increased RR of SCH for all-cause and CVD mortality in the aged group [9]. In this study, relative risk of cancer death in subjects with SCH was not significantly increased in the younger age (less than 65 years old) group. Statistically significant increased adjusted relative risk for cancer deaths in SCH subjects was noted in the older age group. A significant association between SCH and all-cause and CVD mortality in subjects with TSH levels ranging from 5 to 9.9 mIU/l had been reported in our previous study [9]. The present study also revealed a significant association between SCH and cancer deaths in subjects with TSH levels ranging from 5 to 9.9 mIU/l. With relatively small numbers, the results became unstable in subjects with higher TSH levels. Our analysis revealed prominent effects of SCH in cancer deaths among heavy smokers. The importance of quitting smoking should be emphasized in subjects with SCH who are heavy smokers.

Several reports suggested that thyroid hormone supplement might accelerate tumor growth or recurrence [58–59]. Hercbergs et al. suggested prudent consideration for thyroid hormone replacement when managing chemically hypothyroid patients with cancer [57]. To reduce heart failure and CVD mortality, the clinical practice guideline of American Thyroid Association recommends L-thyroxine treatment for patients with serum TSH levels exceeding 10 mIU/L and individualized consideration for SCH subjects with serum TSH less than 10 mIU/L [60]. Our analysis in this study revealed significantly higher cancer mortality in subjects with SCH than euthyroid. The impact of this observation deserves further investigation.

Our analysis demonstrated the association between SCH and risks of cancer mortality. Limitations of this study include: First, measurement of serum total T4 could be influenced by non-thyroidal conditions. We didn’t measure free T4 in our patients. However, measurement of total T4, instead of fT4, was also used in previous reports [8–9]. Second, without thyroid autoantibody data or thyroid sonography, the prevalence of autoimmune thyroid disorders in our participants was not clear. Third, the serum TSH and T4 levels and other laboratory data were checked when subjects were recruited. We did not have follow-up thyroid function data to confirm the persistence of SCH. The changes of other covariates during the follow-up period were also not clear. Furthermore, the thyroxine regimen in SCH patients might potentially affect the risk, progression, or mortality of cancer. We did not know whether the SCH patients were treated by thyroxine or not. Fourth, thyroid dysfunction may have different influence on various cancers. Increasing cancer mortality may be caused by higher cancer incidence, later diagnosis, quality of care, aggressiveness of cancer, or poor response to therapy. Our data was not detailed enough to elucidate the cause of increasing cancer mortality in SCH. Fifth, as an observational study, possible residual confounding factors still remain even after adjustment with demographic, clinical, socioeconomic, and life style factors as in our analysis.

## Conclusions

We have found that SCH was independently associated with an increased risk for cancer mortality among adult Taiwanese.
